# The Many Forms and Functions of Long Term Plasticity at GABAergic Synapses

**DOI:** 10.1155/2011/254724

**Published:** 2011-07-21

**Authors:** Arianna Maffei

**Affiliations:** Department of Neurobiology and Behavior, State University of New York (SUNY), Life Science Building Rm 546, Stony Brook, NY 11794, USA

## Abstract

On February 12th 1973, Bliss and Lomo submitted their findings on activity-dependent plasticity of glutamatergic synapses. After this groundbreaking discovery, long-term potentiation (LTP) and depression (LTD) gained center stage in the study of learning, memory, and experience-dependent refinement of neural circuits. While LTP and LTD are extensively studied and their relevance to brain function is widely accepted, new experimental and theoretical work recently demonstrates that brain development and function relies on additional forms of plasticity, some of which occur at nonglutamatergic synapses. The strength of GABAergic synapses is modulated by activity, and new functions for inhibitory synaptic plasticity are emerging. Together with excitatory neurons, inhibitory neurons shape the excitability and dynamic range of neural circuits. Thus, the understanding of inhibitory synaptic plasticity is crucial to fully comprehend the physiology of brain circuits. Here, I will review recent findings about plasticity at GABAergic synapses and discuss how it may contribute to circuit function.

## 1. Heterosynaptic Inhibitory Plasticity

### 1.1. Long-Term Potentiation

**Figure 1:**
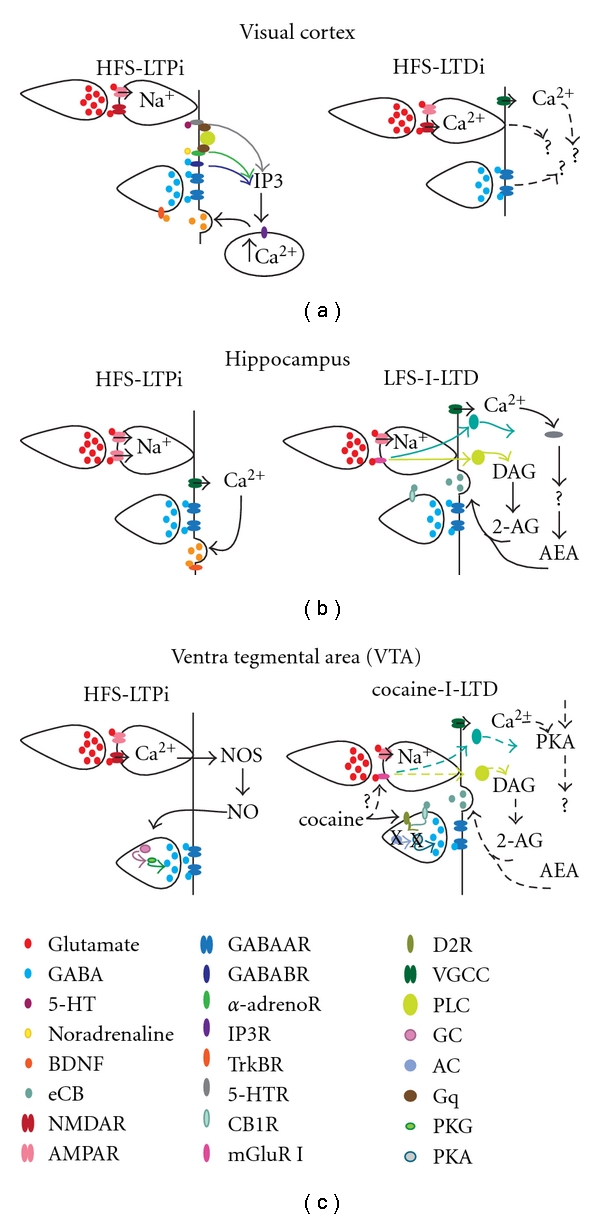
Different mechanisms for heterosynaptic inhibitory plasticity. (a) Visual Cortex. (b) Hippocampus. (c) Ventral Tegmental Area (VTA).

Plasticity of GABAergic synapses onto excitatory neurons, in the form of long-term potentiation (LTPi) and/or depression (LTDi) of inhibitory postsynaptic potentials (IPSPs), was initially reported in layer 5 of the rodent primary visual cortex [1]. Following these pioneering studies, bidirectional inhibitory plasticity was observed in many areas of the brain—neonatal hippocampus [2], deep cerebellar nuclei [3, 4], lateral superior olive [5], brain stem [6], and onto dopaminergic neurons in the ventral tegmental area (VTA) [7, 8]. Although there are significant differences in the induction and expression mechanisms of high-frequency long-term inhibitory plasticity (HF-LTPi and HF-LTDi, Figure 1), some common features have been identified across several brain circuits. Most forms of HF-LTPi involve Ca^2+^-mediated signaling. The source of Ca^2+^ is specific to the inhibitory synapse: voltage-gated calcium channels (VGCC) in neonatal hippocampus (Figure 1(b), left panel) [9]; astrocytes in juvenile hippocampus [10]; postsynaptic intracellular stores in cortex (Figure 1(a), left panel) [11, 12]; activation of postsynaptic NMDA receptors in the VTA (Figure 1(c), left panel) [8]. In several systems, the induction of HF-LTPi and HF-LTDi depends on high-frequency activation of glutamatergic and GABAergic axons. Postsynaptic activation of glutamatergic receptors is often required for the induction of HF-LTPi, while GABA_A_ receptor activity is involved in maintaining the plasticity [1, 8]. By sampling and integrating GABAergic and glutamatergic inputs, heterosynaptic forms of inhibitory plasticity may modulate the dynamic range and output of pyramidal neurons very effectively. 

The intracellular mechanisms involved in the induction and expression of HF-LTPi differ significantly between brain circuits. In visual cortex, Ca^2+^ release from intracellular stores is triggered by the activation of GABA_B_ receptors, facilitated by the activation of serotoninergic (5-HT) and/or *α*-adrenoreceptors [11] and mediated by the activation of IP_3_ [12, 13] (Figure 1(a) left panel). In both developing visual cortex and hippocampus, intracellular Ca^2+^ release initiates a BDNF/TrkB signaling cascade that modulates GABA release [14, 15] (Figures 1(a) and 1(b) left panels). While in the hippocampus, HF-LTPi is induced and maintained after the HFS, in visual cortex the maintenance of HF-LTPi requires constant low-frequency stimulation [16]. The specific mechanisms for this requirement remain to be elucidated.

The mechanisms of HF-LTPi in the VTA are quite different. Although expressed presynaptically as in visual cortex and hippocampus, HF-LTPi in the VTA requires retrograde signaling via a nitric-oxide-(NO-) guanylate cyclase (GC-) protein-kinase-G-(PKG-) dependent pathway [17] (Figure 1(c), left panel). Different mechanisms of induction and expression for HF-LTPi suggest that the specificity of the connection and the patterns of activity may be important for the function of heterosynaptic inhibitory plasticity in different circuits. For example, BDNF retrograde signaling allows for a local action of GABAergic plasticity at specific synapses [14, 18], suggesting a prominent function of HF-LTPi on the local integration of excitatory and inhibitory synaptic events. Differently, at synapses in which production of NO is involved in inhibitory plasticity, the widespread diffusion typical of NO may influence several presynaptic terminals simultaneously [19], possibly promoting changes in the state of excitability of a large portion of a microcircuit. 

### 1.2. Long-Term Depression

The mechanisms for induction and expression of heterosynaptic LTDi show significant differences in different circuits (Figures 1(a), 1(b), and 1(c), right panels). In L5 of primary visual cortex, HF-LTDi is induced by activation of glutamatergic and GABAergic axons and is dependent on Ca^2+^ inflow in the postsynaptic excitatory neuron either through NMDA receptors [1, 13] or through L-type Ca^2+^ channels [20] (Figure 1(a), right panel). The intracellular cascade involved in HF-LTDi is currently not known. It has been speculated that NMDA-dependent HF-LTDi and L-type Ca^2+^channels-dependent HF-LTDi may differ in that the former produces a focal, spatially restricted depression of inhibition, and the latter contributes to depressing many inhibitory synapses onto the same postsynaptic neuron [20]. HF-LTDi (or HF-I-LTD) was also induced in L2/3 of primary visual cortex [21] and in the hippocampus [22]. The mechanisms for these forms of plasticity have been investigated and are known to involve the production of endocannabinoids (eCB) in both L2/3 of visual cortex and hippocampus. In the hippocampus, the production of eCB is dependent on the activation of postsynaptic type I metabotropic receptors (mGluR-I) [22], while in visual cortex the mechanism of activation has not been identified.

A second widely investigated form of heterosynaptic LTDi is induced by low-frequency (LF) activation of glutamatergic axons, which can heterosynaptically depress GABAergic inputs converging onto the activated postsynaptic neuron (LF-LTDi or I-LTD, Figure 1(b)) [23]. This form of plasticity has been reported in several areas of the brain including VTA [24], basolateral amygdala (BLA) [25], dorsal striatum [26], prefrontal cortex [27], and corticotectal cocultures [28]. I-LTD is induced by activation of metabotropic glutamate receptors (mGluR1) and is maintained by postsynaptic GABA_A_ receptors activity. Intracellular pathways activated by the induction of I-LTD lead to the production and the release of endocannabinoids (eCB) from the excitatory neuron [22, 23, 27], which in turn promote changes in strength at inhibitory synapses onto the same postsynaptic target neuron (Figures 1(b) and 1(c), right panels). In the hippocampus, I-LTD requires the activation of inhibitory afferents in the presence of eCB, suggesting that a raise in presynaptic Ca^2+^ in the presynaptic interneuron terminal is required for the induction of this form of inhibitory plasticity [29]. In the VTA, I-LTD is expressed presynaptically and involves a protein-kinase-A-(PKA) dependent modulation of GABA release [22, 30]. Similarly to BDNF-dependent HF-LTPi, eCB-I-LTD signaling is more localized to the area of induction [31, 32]. Given the induction requirements, eCB-I-LTD may contribute to the integration of local associative inputs elicited by long-lasting presynaptic activity in the low-frequency range.

## 2. Homosynaptic Inhibitory Plasticity

### 2.1. Spike-Timing-Dependent Depression of Inhibitory Inputs

**Figure 2:**
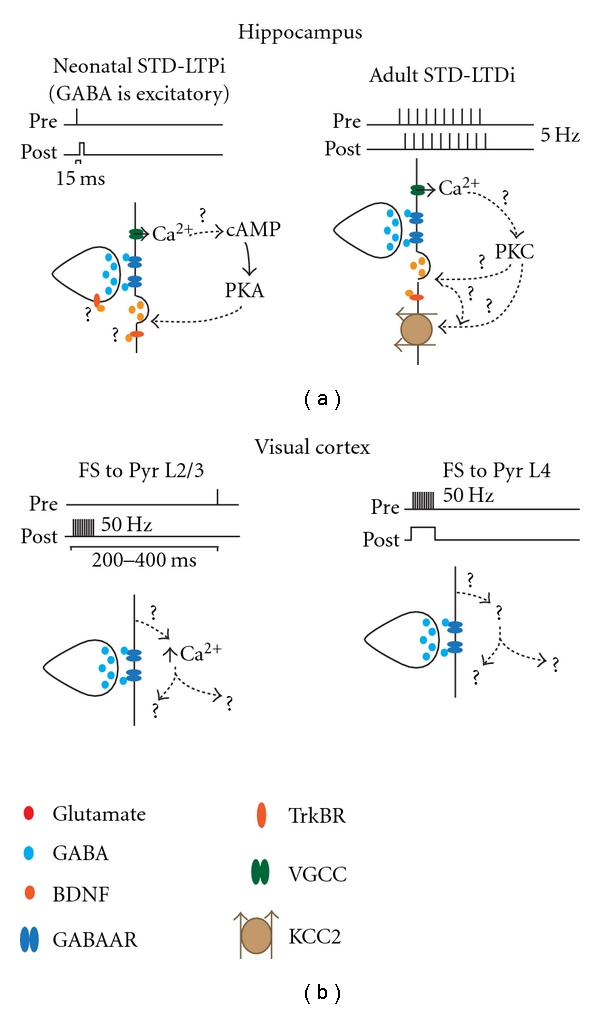
Induction paradigms and mechanisms for monosynaptic inhibitory plasticity. (a) Hippocampus. (b) Visual Cortex.

Homosynaptic forms of inhibitory plasticity have recently been reported in cortex and hippocampus (Figure 2) [15, 18, 33–35]. The patterns of activity reported for homosynaptic inhibitory plasticity differ between brain areas and with the developmental stage of the circuit. Furthermore, homosynaptic monosynaptic forms of inhibitory plasticity have been reported: GABAergic synapses from a single inhibitory neuron of a specific subtype onto a postsynaptic excitatory neuron can change their strength in response to patterned stimulation [34, 35]. GABAergic synapses may modulate their strength not only to regulate the integration of excitatory and inhibitory inputs, but also in response to a variety of input patterns, possibly increasing the range of functions that inhibitory plasticity may perform in different circuits. 

In immature hippocampus, when GABA is excitatory, stimulations eliciting action potential firing in afferent axons 15 ms before postsynaptic firing potentiate GABA postsynaptic currents onto CA3 pyramidal neurons (Figure 2(a), left panel), while the opposite timing relationship induces a consistent depression of GABA postsynaptic current amplitudes [18]. The mechanisms leading to spike-timing-dependent long-term potentiation of inhibition (STD-LTPi) have been further investigated, showing that this form of GABAergic plasticity is induced postsynaptically and expressed presynaptically [18]. The signaling pathways involved in STD-LTPi in neonatal hippocampus depend on the increase in postsynaptic Ca^2+^ levels and on the activation of a cAMP-PKA intracellular pathway. STD-LTPi required retrograde BDNF signaling (Figure 2(a), left panel) [18], consistent with reports that postsynaptic backpropagating action potentials trigger the release of BDNF in the postsynaptic neurons [15]. The site of action of BDNF is at the moment unclear, as postsynaptic [15] and/or presynaptic [36] actions of the BDNF/TrkB signaling pathway have been reported. STD-LTPi in the immature hippocampus increases the activity of excitatory neurons and is thought to contribute significantly to the development and refinement of hippocampal circuits [37, 38]. 

In adult hippocampal slices, when GABA is hyperpolarizing and exerts an inhibitory action, homosynaptic plasticity of GABAergic synapses is successfully induced when presynaptic action potentials are elicited at inhibitory axons coincidentally with the generation of postsynaptic action potentials in CA1 pyramidal neurons (STD-long-term depression of inhibition, STD-LTDi: Figure 2(a), right panel) [33, 39–41]. STD-LTDi induction depends on postsynaptic Ca^2+^ influx through voltage-gated Ca^2+^ channels and on the depolarization of the equilibrium potential for chloride by the neuron-specific chloride extruding transporter KCC2 [33] (Figure 2(a), right panel). Interestingly, activation of the BDNF-TrkB pathway regulates the levels of expression of KCC2 in the adult hippocampus [42], suggesting possible complementary mechanisms of expression for HF-LTPi in cortex [14] and STD-LTDi in the adult hippocampus [33] (Figure 2(a)). In HF-LTPi the high-frequency stimulation may promote a BDNF-dependent increase in inhibitory synaptic conductance through modulation of presynaptic release probability (Figure 1(b), left panel) [14], while the lower frequency of stimulation required by STD-LTDi induction may favor BNDF-dependent downregulation of the KCC2 transporter [42]. 

### 2.2. Pre- and Postsynaptic Pairing in Homosynaptic-Monosynaptic Inhibitory Plasticity

Timing is a fundamental feature of homosynaptic inhibitory plasticity; however, there are substantial differences in the requirements for induction and expression of GABAergic homosynaptic plasticity in hippocampus and sensory neocortex. In visual cortex in particular, homosynaptic inhibitory plasticity has been studied at monosynaptic connections from identified inhibitory neurons onto pyramidal neurons. Therefore, the plasticity I will describe in this paragraph is both homosynaptic—it depends on the activation of inhibitory axons alone—and monosynaptic—it is induced by activation of connections mediated by a single axons from an indentified inhibitory neuron subtype onto a pyramidal neuron.

In layer 2/3 of visual cortex, the sign of plasticity at inhibitory synapses from fast spiking (FS) onto pyramidal neurons depends on the timing between presynaptic FS neuron firing and postsynaptic pyramidal neuron bursting [35]. Burst timing LTD (BT-LTDi) is induced if FS action potentials are elicited 100 ms after pyramidal neuron bursting, while BT-LTPi is induced when FS firing was elicited 200 to 300 ms after postsynaptic bursting (Figure 2(b), left panel). Both BT-LTPi and BT-LTDi depend on postsynaptic calcium influx, although the source of such increase has not been determined [35]. Differently from the forms of inhibitory plasticity reported in L5 of primary visual cortex or in the hippocampus, BT inhibitory plasticity does not appear to require GABA_B_ receptor activation, activation of NMDA receptors, or changes in KCC2 activity [35] (Figure 2(b), left panel), suggesting that this form of inhibitory plasticity may rely on a set of mechanisms yet to be identified. 

Although timing of pre- and postsynaptic activity is indeed a general feature of FS to pyramidal neuron inhibitory plasticity, the requirements for timing and patterns of activity differ significantly, even between layers of primary visual cortex. In Layer 4, potentiation of FS to pyramidal neurons synapses (LTPi) requires coincident activation of FS and postsynaptic pyramidal neurons, but FS interneuron bursting needs to be paired with pyramidal neuron subthreshold depolarization [34] (Figure 2(b), right panel). Despite the differences, both BT-LTPi and LTPi are expressed as changes in the conductance of inhibitory synapses and appear to have a postsynaptic site of expression, possibly depending on an increase in GABA_A_ R number [34, 35, 43]. Both BT-LTPi and LTPi are induced using paired recording experiments, indicating that these forms of plasticity are specific to the interneuron type [34, 35]. The cellular mechanisms for BT-LTPi and LTPi are currently unknown. Several intracellular pathways have been reported to regulate the number of GABA_A_ receptors at inhibitory synapses [44–47]. An intriguing possibility is that some of these mechanisms may be also involved in the fast transport required for plasticity. 

A different subset of inhibitory synapses in neocortex, the ones from low-threshold spiking interneurons (LTS) onto spiny stellate neurons in the barrel cortex and from regular spiking non-pyramidal neurons (RSNP) onto pyramidal neurons in visual cortex, show modulation of synaptic efficacy in response to changes in circuit excitability [48, 49]. In visual cortex, reduction of visual drive right at eye opening strengthens their synapses onto pyramidal neurons, decreases their connection probability, and leaves their short-term dynamics unaffected [49]. In barrel cortex, LTS neurons—also classified as SOM neurons—change their short-term dynamic [48] and intrinsic properties [50] in response to activity blockade. The induction and expression requirements for plasticity at these inhibitory synapses have yet to be identified. 

Overall there is richness in the forms of inhibitory plasticity and in the variety of mechanisms involved in their induction and expression in different areas and at different developmental stages. This evidence suggests that inhibitory synapses may have important and highly specific functions that contribute to the control of the excitability of neural circuits in complex ways.

## 3. Functional Implications of Inhibitory Plasticity

### 3.1. Maintenance of Circuit Stability and Circuit Refinement

A number of studies have shown that inhibitory synaptic transmission is crucial for the development [51–53] and stability of neural circuits [54, 55], sharpens tuning of principal excitatory neurons [56–59], and contributes to the formation of receptive fields [60, 61]. All of these functions are based on the assumption that inhibitory synapses are not plastic and exert what is thought to be their principal task: suppress excessive excitability [62] and possibly increase the signal-to-noise ratio [63]. 

It is now clear, however, that inhibitory synapses are indeed plastic. What is the role of this plasticity? The ability to directly and dynamically control pyramidal neuron input integration, excitability, and output in an activity-dependent manner, suggests that inhibitory synaptic plasticity may be crucial to preserve the dynamic range of excitatory neurons [64] even when the excitability of the circuit is perturbed by changes in environmental inputs [49]. The maintenance of circuit stability is indeed a dynamic process that requires plasticity of many cellular and synaptic components of a neural circuit [65, 66].

The richness in plasticity and the specificity of inhibitory circuits suggest that the dynamic regulation of circuit homeostasis is not the sole function of inhibitory plasticity. In primary sensory areas, incoming inputs regulate the maturation of GABAergic transmission, promote the refinement of the connectivity of local microcircuits, and regulate the overall excitability of the circuit [21, 34, 49, 67–70]. The sharpening of cortical receptive fields during development is also temporally correlated with the maturation of inhibitory synapses [71], and the modulation of excitability by dynamic adjustment of the balance between excitation and inhibition favors the refinement of neuronal receptive fields [72, 73]. At the network level, the regulation of inhibitory synaptic efficacy through plastic changes may contribute to the formation and/or rearrangement of cortical maps [71]. 

Specific inhibitory circuits may contribute differently to the refinement process, as suggested by the effects that paradigms of sensory deprivation produce on the two major populations of inhibitory neurons [48–50]. In rodent neocortex, both the barrel field of somatosensory cortex and primary visual cortex show depression of FS synaptic inhibition onto pyramidal neurons in response to sensory deprivation during early postnatal development [48, 49]. FS to pyramidal neuron synapses receive direct thalamocortical projections [74, 75] and,thus, are in a particularly favorable anatomical position to convey information about changes in sensory inputs. A possible function of reduced FS inhibition at this stage in development is to preserve the overall state of excitability of the circuit in the face of a reduced driving input. In primary visual cortex, evidence in favor of this hypothesis comes from the lack of ocular dominance shifts following visual deprivation between eye opening and the beginning of the classical critical period for amblyopia [76]. In the barrel cortex, the decrease in somatic inhibition might play a similar homeostatic role [48].

### 3.2. Regulation of Circuit Function

The same inhibitory synaptic connection may play different functions during different stages in postnatal development. After the third postnatal week, instead of weakening FS to pyramidal neurons synapses, visual deprivation induces LTPi of these synapses in monocular cortex [34] and a general potentiation of inhibitory drive in binocular visual cortex [77]. The switch in sign of MD-induced inhibitory plasticity correlates with the time of initiation of the critical period for ocular dominance plasticity [76, 77]. A possible interpretation of these results is that as the visual cortex matures, the role of inhibitory plasticity changes, going from regulator of global circuit homeostasis to driver of activity-dependent circuit refinement. LTPi may contribute to the silencing of neurons driven by the deprived eye, possibly favoring the shift in ocular dominance to the eye that remained open. To perform these functions, LTPi should be connection-specific, regulating the excitability of excitatory neurons only within local microcircuits. In addition, it should have an effect on the sign of plasticity at excitatory synapses. While the role of inhibition in controlling excitatory neurons excitability is widely accepted, the other properties still need to be investigated experimentally. 

Besides modulating FS to pyramidal neurons synapses, visual deprivation significantly affects another inhibitory connection in neocortex: the one from RSNP and LTS interneurons onto pyramidal neurons. Similar paradigms of sensory deprivation modulate the strength of RSNP and LTS inhibitory synapses onto excitatory neurons [42]. It is tempting to speculate that environmental stimuli or behaviors may modulate the strength of inhibitory synapses in ways that are specific for the type of inhibitory neuron and favor different circuit-rewiring patterns. 

LTS and RSNP inhibitory neurons contact the apical dendrites of pyramidal neurons while FS synapses are found at the soma and proximal dendritic shafts [78]. Plasticity at LTS and RSNP synapses may be involved in the modulation of the local integration of distal inputs, while FS synapses regulate the integration of all inputs reaching the soma. The integration of all inhibitory and excitatory inputs shapes the state of excitability of a circuit throughout life; thus, the contribution of inhibitory plasticity to neural circuit function is likely to extend beyond circuit refinement. The dynamic regulation of the balance between excitation and inhibition that is induced by changes in inhibitory and excitatory synaptic strength may affect the coding of specific sensory stimuli and serve as an important mechanism for cortical sensory processing throughout life.

### 3.3. Beyond Sensory Function

Beyond sensory cortices, inhibitory plasticity is induced in circuits involved in learning and addictive behaviors [7, 8, 15, 22, 33, 79, 80]. Indeed, in the VTA, HF-LTPi is impaired by morphine exposure [8], and I-LTD is favored by repeated cocaine exposure [24]. These forms of plasticity alter the activity of dopaminergic neurons in the VTA following drug abuse, possibly facilitating the development of addictive behaviors [81]. In the VTA, other forms of inhibitory plasticity have been reported that do not require patterned activation of afferent fibers but are dependent on the administration of drug of abuse [80, 82, 83] and appear to be facilitated by coactivation of serotoninergic receptors [82]. It is currently unknown whether the administration of a drug of abuse and the patterned activation of neurons may activate convergent of cellular targets or whether the electrical and chemical inhibitory plasticity are distinct processes. 

Inhibitory plasticity in the VTA was also proposed as mechanism for metaplasticity [81], a way to constrain or change the state of a postsynaptic neuron to limit or direct the plasticity at other synapses converging onto it [84]. While there is no clear experimental evidence for a metaplastic role of inhibitory plasticity, it would certainly add to the already complex set of functions this plasticity appears to perform.

In neonatal hippocampus, when GABA is excitatory [85], the role of GABAergic plasticity may be to promote circuit wiring and maturation by activating pyramidal neurons [18, 86]. In the adult hippocampus, STD-LTDi modulates the output and the dynamic range of pyramidal neurons in CA1 globally by shifting the reversal potential of chloride of the postsynaptic neuron [39, 40]. In addition, it was recently suggested that STD-LTDi regulates the effectiveness of backpropagating action potentials [64], suggesting that inhibitory plasticity may be a modulating mechanism for the induction of LTP or LTD at synapses between excitatory neurons. A more focal regulation of specific inputs onto excitatory neurons may be performed locally, at specific synapses, by heterosynaptic HF-LTPi and BDNF-Trkb signaling [15]. Both global and synapse-specific inhibitory synaptic plasticity likely regulate the local integration of incoming inputs onto pyramidal neurons in a complementary fashion, possibly favoring or impairing the induction of other forms of plasticity.

## 4. Future Directions

Although the study of inhibitory plasticity started a couple of decades ago, it has had a fast and steady growth only recently. Besides regulating the activity and computation of local microcircuits in many areas of the brain, inhibitory plasticity has been implicated in sensory processing [61], in the learning of sound localization [87], in the regulation of neuropathic pain [88], in the regulation of neural activity following brain injury [89, 90], as well as in changes in neuronal excitability induced by pregnancy [91]. Much work is needed to identify mechanisms as well as targets for the selective manipulation of GABAergic synaptic plasticity in different brain circuits. The findings that specific inhibitory neuron subtypes contact excitatory neurons at different locations [92, 93] and that the postsynaptic membranes opposite to the different subtypes of interneurons contain GABA_A_ receptors with specific subunit composition [94–97] offer remarkable tools to jump start this investigation. The compelling data about locations, range of induction and expression mechanisms, specificity, and associativity, together with the functional implications of inhibitory synaptic plasticity, strongly support the idea that plasticity at GABAergic synapses is a fundamental regulator of the physiology of neural circuits. Advancements in our understanding of the different forms of inhibitory plasticity are crucial to address more directly their many roles in healthy brain function and disease.
